# Cost and impact of decentralized tuberculosis testing: a modeling analysis of price thresholds for molecular instruments in high-burden settings

**DOI:** 10.1016/j.eclinm.2025.103728

**Published:** 2025-12-29

**Authors:** Tom Ockhuisen, Alexandra de Nooy, Sarah Girdwood, Megan A. Hansen, Mikashmi Kohli, Morten Ruhwald, Nazir Ismail, Brooke E. Nichols

**Affiliations:** a Department of Global Health, Amsterdam Institute for Global Health and Development, Amsterdam UMC, University of Amsterdam, Amsterdam, Netherlands; b FIND, Geneva, Switzerland; c Department of Clinical Microbiology and Infectious Diseases, Faculty of Health Sciences, University of the Witwatersrand, Johannesburg, South Africa; d Department of Global Health, Boston University School of Public Health, Boston, MA, US; e Wits Diagnostic Innovation Hub, Faculty of Health Sciences, University of the Witwatersrand, Johannesburg, South Africa

**Keywords:** Tuberculosis, Diagnostics, Health economics, Decentralized testing

## Abstract

**Background:**

Despite progress in reducing global tuberculosis (TB) incidence, ongoing funding disruptions threaten to reverse gains. Timely, accurate diagnosis integrated with rapid treatment is critical to reducing morbidity, mortality, and transmission—but remains costly, particularly for decentralized approaches. As global health budgets tighten, identifying the optimal cost for molecular TB diagnostics instruments is critical to inform scale-up decisions.

**Methods:**

We developed a probabilistic patient pathway model to assess the cost-effectiveness of decentralizing TB testing across primary healthcare facilities in high-burden settings. Scenarios varied by facility testing density (low, medium, high) and anticipated testing volume increases (none, partial, full). We included the effects of instrument downtime and calculated the maximum instrument + warranty prices at which fully decentralized testing would be considered cost-effective at a $500/disability-adjusted life year (DALY) averted threshold.

**Findings:**

Decentralization with increased testing averted up to 23% more DALYs than centralized testing but was not cost-effective at current instrument + warranty prices. Partial decentralization to the largest 20% of facilities approached cost-effectiveness (<$1000/DALY) with full testing increase. For full decentralization to meet the $500/DALY threshold, maximum viable instrument + warranty prices for low-throughput instruments ranged from $0 (no uptake increase) to $410–$6048 (increased testing uptake, low- to high-testing density settings).

**Interpretation:**

At current prices, decentralized molecular TB testing is unlikely to be cost-effective, even with improved uptake, systemwide. However, meaningful reductions in instrument + warranty costs could make both full and partial decentralization viable. As countries face tighter budgets, clear price targets for cost-effectiveness can help guide procurement and investment decisions in TB diagnostics.

**Funding:**

10.13039/100000865BMGF, Willem Bakhuys Roozenboomstichting.

**Research in context**

**Evidence before this study**

Tuberculosis (TB) remains a global health threat and causes the greatest number of deaths due to infectious disease. Despite significant efforts to reduce incidence and mortality, through finding and treating TB, a large diagnostic gap remains, stemming from limited access to accurate, timely, and affordable diagnostics. Decentralization of molecular TB testing is one potential strategy to increase access. We searched PubMed using the search terms “TB diagnostics”, “TB test (ing)”, “decentralization”,” molecular tests”, and “cost-effectiveness” for studies published before April 2025. Prior work has evaluated decentralization of GeneXpert instruments for TB and other molecular diagnostics (e.g., HIV viral load) in multiple countries. These studies highlighted instrument utilization as a key driver of cost-effectiveness, as underused instruments markedly increase costs with little gain in test volumes. They also recognized trade-offs between decentralized and centralized testing, noting that decentralization may cost less than building sample referral systems, though few considered shared infrastructure across programs. Several studies found decentralization of GeneXpert for TB diagnosis to be cost-effective or cost-saving under certain assumptions, but most were country-specific, trial-based, and did not evaluate system-wide cost-effectiveness or optimal instrument pricing.

**Added value of this study**

This study extends previous work by evaluating both the cost-effectiveness and viable instrument prices for decentralized TB testing across a whole health system. The analysis is country-agnostic, considers different facility testing densities and demand increases, and explicitly incorporates instrument downtime. Unlike earlier trial-based or single-volume analyses, our model simulates a distribution of volumes across facilities to better reflect real-world implementation. We show that decentralization averts additional disability-adjusted life years (DALYs) by increasing the number of people diagnosed, but is unlikely to be cost-effective if testing demand does not simultaneously rise with decentralization. Partial decentralization to high-volume facilities could approach cost-effectiveness if demand increases and downtime is minimized. This is the first study, to our knowledge, to provide explicit target price ranges ($0–$6048 for low-throughput) for instruments to make decentralization feasible and cost-effective.

**Implications of all the available evidence**

Decentralized molecular testing could help close the diagnostic gap by improving access to timely results and treatment initiation, thereby reducing DALYs. However, at current instrument prices, full decentralization is unlikely to be cost-effective because of the large number of devices required. Partial decentralization focused on high-volume facilities emerges as a more viable strategy in the short term with currently available instruments, especially if demand increases. These findings are broadly consistent with prior country-level studies, such as those by Sohn et al., Malhotra et al. (TB-CAPT trial), and Thompson et al. (XPEL-TB study), which all highlighted utilization as a central determinant of cost-effectiveness. By combining a health system-wide perspective with explicit instrument price targets, our analysis provides actionable guidance for policymakers and donors. When combined with in-country knowledge of infrastructure and demand, these results can help inform procurement and investment decisions for TB diagnostic programs.

## Introduction

Despite reductions in global tuberculosis (TB) incidence over the past decade, current global health funding disruptions further threaten the progress made in controlling TB globally.^1^ Effective reduction in the global TB burden requires improvements in both the timeliness and accuracy of diagnostic strategies, alongside systems to ensure linkage to appropriate treatment. Access to accurate and timely diagnosis, integrated with prompt treatment initiation, reduces morbidity and mortality while interrupting transmission dynamics—key factors for achieving epidemic control.^2^^,^^3^

Widescale implementation of molecular WHO-recommended rapid diagnostics for screening (mWRDs) is critical to achieving the first step in the TB care cascade: accurately diagnosing individuals. In 2010, the World Health Organization (WHO) recommended the use of GeneXpert MTB/RIF as the primary diagnostic tool in high-burden countries for individuals presenting for care with signs and symptoms suggestive of TB.^4^ Although molecular TB diagnostics outperform previously used diagnostics (e.g., sputum smear microscopy (SSM)), the requirements for trained staff and infrastructure combined with the high capital costs of molecular instruments have led to implementation and scale-up challenges in many high-TB-burden countries.^5^, ^6^, ^7^ Of the 10.8 million people estimated to have TB in 2023, only 3.9 million were diagnosed with mWRDs.^8^

Many high-TB-burden countries have implemented centralized molecular testing, either through a ‘hub-and-spoke’ model where specimens are transported to testing facilities, or by referring individuals to testing sites to enable testing with more accurate diagnostics.^9^ In a centralized testing model, molecular instruments are installed at designated facilities that possess adequate security, power supply, and the necessary infrastructure to function as collection hubs to process samples provided by lower tier facilities.^10^

While the centralized model for molecular TB testing represents a significant improvement over prior strategies, decentralization of molecular diagnostics may further improve access to TB testing.^11^ Through decentralization, molecular testing would be available on-site, and depending on its implementation, could provide results to the individual before leaving the healthcare facility (enabling same-day test and treat^2^^,^^3^). Therefore, decentralized testing can increase the number of people correctly diagnosed by reducing specimens lost through transport and minimizing potential loss to follow-up (LTFU) by providing same-day results. Decentralized testing has also been shown to increase demand for testing while simultaneously increasing the ‘diagnostic yield’ or the total number of people correctly diagnosed with TB.^11^^,^^12^ Decentralization of TB testing has also been broadly demonstrated to be cost-effective, or even cost-saving in a number of settings.^13^, ^14^, ^15^ Decentralization, however, comes with potential drawbacks. When relying on a single instrument per site, sites may lose access to testing during any instrument downtime (downtime defined as instruments being out of service for any reason)—an average of 30 days per year.^7^ For instrument breakdowns (rather than power supply issues), technicians are dispatched to facilities to conduct repairs or maintenance. However, access to timely repairs can be challenging in remote locations due to logistical constraints. Downtimes are likely to persist in conjunction with increased required servicing of more instruments. Thus, the cost of truly decentralized testing has been shown to be prohibitively high, with the cost of a molecular diagnostic instrument often driving this cost due to low instrument utilization.^13^^,^^16^

To assess the maximum potential impact of currently available molecular diagnostics for TB, we evaluated the resource requirements, health outcomes, and cost-effectiveness of varying levels of decentralized molecular testing compared with a fully centralized model across a range of healthcare system archetypes. Given that instrument utilization,^13^^,^^16^ and thus, in effect, instrument price, drives cost-effectiveness, we further estimated the instrument price at which fully decentralized testing would become cost-effective across healthcare systems with different densities of TB testing. This analysis was conducted in a country-agnostic framework to ensure broad relevance across high-TB-burden settings.

## Methods

We developed an algebraic mathematical model to understand the potential cost-effectiveness of differing levels of decentralization of TB testing in primary healthcare facilities (an individuals’ first point of contact in the healthcare system) across a range of healthcare settings. The model developed for this analysis incorporates outputs from our previously published patient pathway model of TB diagnosis at different levels of healthcare.^17^ Several factors were included in this analysis, namely: specimen transport costs, expected LTFU throughout the diagnostic cascade and expected downtime of instruments. We have benchmarked our primary analysis of cost-effectiveness to the GeneXpert instrument + warranty costs, as currently these are the instruments primarily employed for molecular TB diagnosis in most countries.^18^ We then varied costs and assumptions to identify the cost at which molecular instruments would become cost-effective for full- and partial-decentralization. Unless otherwise specified, references to molecular testing are intended to be platform-agnostic, while acknowledging that several assumptions are based on GeneXpert due to the availability of data. The model was developed in Microsoft Excel (Microsoft Corporation, Redmond, WA, USA; version 16.99.2).

### Estimation of TB tests per healthcare facility

The cost per molecular TB test is strongly linked to utilization of an instrument. With low utilization, the cost per test is high given the cost of the instrument is divided by a small number of tests.^19^^,^^20^ We assumed that 500,000 tests were performed annually and that these tests were done in the context of a healthcare system that had either 1000 (high-density testing), 2000 (medium-density testing), or 5000 (low-density testing) healthcare facilities. The average number of tests per facility per year range from 500 (high-density) to 100 (low-density), roughly reflecting the ranges observed across many high-TB-burden countries (Table S2). We employed a Pareto distribution in each density distribution of healthcare facilities, given that a small number of facilities typically contribute to a disproportionate share of the total number of tests.^21^ This distribution is characterized by a heavy tail, effectively capturing the skewed nature of test volumes across healthcare facilities in high-burden settings.^22^^,^^23^ From the annual estimated TB tests per facility, we estimate a daily number of TB tests performed utilizing 260 working days per year. With this estimate, we allocate appropriately sized instruments (which can do an assumed 6, 12, and 48 tests per day at 75% utilization^20^^,^^24^) to each facility accommodating peak testing volumes for our baseline analyses and ensuring same-day results.

### Demand increase assumptions

Decentralization of testing has been demonstrated to affect overall testing demand in healthcare settings, likely stemming from increased diagnostic access. Reported findings suggest these increases could be up to 90%.^11^ As such, all analyses have been replicated for three assumptions of changed test access through decentralization: 0% (no increase), 45% (partial increase) and 90% (full increase).

### Model description and health outcomes estimation

The probabilistic patient pathway model is initialized by assigning a cohort of 500,000 symptomatic individuals needing diagnosis, who then progress through the diagnostic pathway until diagnosis. To estimate the number of positive test results from the total daily TB tests performed at each facility, we multiplied the total number of tests conducted by a test positivity rate, which was either centralized (11%) or decentralized (8%).^11^ The 8% test positivity rate was used only when decentralization was assumed to increase testing volume.

Through decentralization of molecular testing, potential LTFU is minimized. Centralized testing involves referring specimens through a specimen transport system (where specimens could be lost) or referring individuals to another testing site (where they may not seek care). For decentralized testing, individuals may receive results during their visit, obviating the need to return, and increasing the likelihood of receiving their result. In this study, we only considered sputum as a sample type (current molecular testing standard). To capture these differences in diagnostic pathways, we varied endpoint values for notification rate of individuals with TB: centralized (84%), decentralized (86%), and decentralized same-clinical-encounter (89%). These values reflect the diagnostic cascade and the test sensitivity (85%) and were estimated through our recently published model which accounted for loss at each step in the cascade, with results averaged across the three countries used in that primary analysis (Kenya, South Africa, India).^17^

We evaluated the costs and expected outcomes of one fully centralized, two partially decentralized, and two fully decentralized operational models (Table 1, full description Text S1).

**Table 1:** Description of operational models for centralized, partly decentralized, and fully decentralized testing.

Operational model	Operational model type	Same-clinical-encounter results^a^	Description
1	Fully centralized	No	All healthcare facilities transport samples to a centralized facility
2	Partly decentralized	No	The 20% largest healthcare facilities test on-site. The smallest 80% transport samples to a centralized facility^b^
3	Partly decentralized	Yes	The 20% largest healthcare facilities test on-site with same-clinical-encounter results. The smallest 80% transport samples to a centralized facility^b^
4	Decentralized	No	All healthcare facilities test on-site
5	Decentralized	Yes	All healthcare facilities test on-site with same-clinical-encounter results

Operational models vary based on the proportion of healthcare facilities conducting on-site testing and whether same-clinical-encounter results are provided. Centralized testing involves transporting all samples to a central facility, while decentralized models can increase on-site testing capacity.

a Loss to follow-up considered for scenarios where same clinical encounter results do not occur.^17^

b The largest and smallest facilities are a reflection of the testing volume, not the actual physical size of the facilities.

### Cost and cost-effectiveness analysis

Cost inputs in the model estimated the daily cost per operational model and evaluated trade-offs between models. All costs are reported in 2024 USD and adjusted for inflation using the Consumer Price Index inflation calculator from the World Bank.^25^ The main cost components are described in Text S2, Table S3, and Table S4. Our analysis adopts a healthcare provider's perspective over a 1-year time horizon of full implementation.

While our modeling framework allows for direct estimation of cases detected, we have furthered this to link cases detected to disability-adjusted life years (DALYs) averted leveraging previous literature. The literature estimates that approximately one DALY is averted (over a 10 year time horizon, discounted at 3% per year) per additional diagnosis.^26^^,^^27^ This is assumption is based on an interpretation of Azman et al.,^26^ which estimated a range of DALYs averted between 0.86 and 1.41 depending on country context over a 10 year time horizon discounted at 3% per year, and was interpreted by Brümmer et al.^27^ to be approximately 1 DALY on average (note: uncertainty analysis, described below, incorporates the full range of uncertainty). Net DALYs averted are assigned to all diagnoses that occur in the 1 year of full implementation within the model. In doing this, we are able to estimate cost per DALY averted under different diagnostic configurations to enable the threshold analyses described below. Incremental cost-effectiveness ratios (ICERs) were compared against a cost-effectiveness threshold of $500 per DALY averted.^28^

### Threshold and sensitivity analysis

A series of threshold analyses were conducted to identify the instrument + warranty price point at which fully decentralized testing would be considered cost-effective (operational model 5), using a threshold of $500 per DALY averted. Understanding this price point is critical as many new diagnostics are being developed, thus a realistic price point for decentralized testing instruments is essential for guiding implementation in high-TB-burden settings.

The analyses explored six scenarios (Table 2) that varied two factors: 1) the assumed change in testing demand if on-site testing were available and 2) the inclusion or exclusion of instrument downtime for decentralized testing. Downtime for decentralized testing accounts for potential operational challenges, such as instruments being non-functional, resulting in missed testing opportunities. This contrasts with centralized sites which are often equipped with multiple instruments, or can store specimens if an instrument is down, mitigating interruptions. Downtime is assumed to be an average of 30 days per year.^7^

**Table 2:** Description of scenarios evaluated: changes in testing demand and downtime inclusion.

Scenario	Increase in testing demand due to decentralization	30-day downtime included
1	0%–no increase	No
2	0%–no increase	Yes
3	45%–partial increase	No
4	45%–partial increase	Yes
5	90%–full increase	No
6	90%–full increase	Yes

To assess the robustness of our conclusions, we conducted a sensitivity analysis of the average cost per DALY averted under two full decentralized testing scenarios: (1) partial testing increase with a low-density testing distribution, and (2) partial testing increase with a high-density testing distribution. We also evaluated the incremental cost per DALY averted to examine how changes in assumptions affected not only the average cost-effectiveness but also the relative position of each scenario on the cost-effectiveness frontier. Seven key input parameters were varied in this analysis: DALYs per person diagnosed (0.5–1.92), test positivity when expanding current testing (from the reduced-prevalence assumption to the baseline of 11%), prevalence among those tested (5%–15%), price of test consumables ($3–$15), cost per minute of laboratory worker time ($0.03–$0.15), cost per minute of healthcare worker time ($0.03–$0.15), and peak testing throughput (doubling the number of individuals presenting for testing at a single time).

### Informed consent

Informed consent was not required for this work. This manuscript represents a modeling analysis that did not require any additional data collection or participant interaction.

### Ethics statement

No ethics approval was required for this work.

### Role of the funding source

Funding provided by The Bill and Melinda Gates Foundation (INV-045721) and the Willem Bakhuys Roozeboomstichting. The funder of the study had no role in study design, data collection, data analysis, data interpretation, or writing of the report.

## Results

### Cost and cost-effectiveness of decentralization

The cost per test was estimated across the five operational models and three facility density distributions under the assumption of no change in testing demand (Table 3, Table S5). For a high-density testing distribution, the total cost per test ranged from $17.79 for centralized testing (operational model 1) to $21.80 for fully decentralized testing (operational model 5)—a 23% increase in cost per test. For medium and low-density testing distributions, the total cost per test increased to $28.56 and $49.16, respective increases of 61% and 176%. Under current instrument + warranty price assumptions, equipment costs increased substantially with decentralization and composed an increasing proportion of these costs. In a high-density testing situation, equipment costs ranged from $1.35 per centralized test (8% of total cost) to $8.74 per tests for fully decentralized testing (40% of total cost). For a more dispersed healthcare system with low-density testing, equipment costs were $36.10 per test when testing was fully decentralized (73% of total test cost). Similar cost trends were seen under differing assumptions of test demand (0%, 45% or 90% increase), although the proportional cost increases became smaller as test demand increased (Appendix A, Appendix A).

**Table 3:** Cost per test (2024 USD) across different facility density distributions for key operational models.

Testing density- facility distribution	Operational model 1	Operational model 2	Operational model 3	Operational model 4	Operational model 5
High-density	$17.79	$17.68	$18.98	$20.66	$21.80
Medium-density	$17.79	$18.13	$19.29	$27.49	$28.56
Low-density	$17.79	$21.50	$22.56	$48.14	$49.16

Costs increase with greater decentralization and same-clinical-encounter results (models 3 and 5), reflecting higher operational and equipment costs compared to centralized and partly decentralized operational models. The full breakdown of cost categories is provided in Tables 1–3.

Regarding health outcomes, decentralized testing performs slightly worse than centralized testing (Table 4). However, with a partial testing increase (45%), decentralized testing averts 9% more DALYs compared to centralized testing. This difference is greatest for a full testing increase, where decentralized testing averts 23% more DALYs than centralized testing.

**Table 4:** Daily costs, disability adjusted life-years (DALYs) averted and incremental cost-effectiveness ratios (ICERs) across key operational models, facility density distributions and various testing uptake scenarios (no testing increase, partial increase, full increase) and a 30-day instrument downtime.

No testing increase				Partial testing increase				Full testing increase			
Operational model	Cost (daily)	DALYs averted (daily)	ICER	Operational model	Cost (daily)	DALYs averted (daily)	ICER	Operational model	Cost (daily)	DALYs averted (daily)	ICER
High-density testing distribution											
1	$34,212	179	$17	1	$34,212	179	Ref	1	$34,212	179	Ref
2	$34,006	167	Ref^a^	2	$43,007	184	WD	2	$52,009	201	WD
3	$36,509	172	D	3	$46,116	190	$1074	3	$55,723	208	$746
4	$39,723	162	D	4	$52,156	187	D	4	$64,589	211	WD
5	$41,931	169	D	5	$55,459	195	$1960	5	$68,987	220	$1069
Medium-density testing distribution											
1	$34,212	179	Ref	1	$34,212	179	Ref	1	$34,212	179	Ref
2	$34,871	165	D	2	$45,053	185	WD	2	$55,235	205	WD
3	$37,097	171	D	3	$48,353	192	$1095	3	$59,610	212	$756
4	$52,874	162	D	4	$65,225	187	D	4	$77,575	211	D
5	$54,930	169	D	5	$68,338	195	$6811	5	$81,747	220	$2898
Low-density testing distribution											
1	$34,212	179	Ref	1	$34,212	179	Ref	1	$34,212	179	Ref
2	$41,337	164	D	2	$52,565	186	WD	2	$63,792	208	WD
3	$43,374	170	D	3	$55,652	193	$1480	3	$67,928	216	$894
4	$92,575	162	D	4	$104,882	187	D	4	$117,189	211	D
5	$94,538	169	D	5	$107,888	195	$38,323	5	$121,238	220	$15,024

D- dominated (more costly and averts fewer DALYs than the next least costly scenario on the cost-effectiveness frontier).

WD- weakly dominated (not on the cost-effectiveness frontier).

a Reference in this block is different, as operational model 2 is least costly.

When assessing cost-effectiveness, no decentralized operational models were considered cost-effective at a cost-effectiveness threshold of $500 per DALY averted regardless of testing uptake and facility distributions (Table 4). When assuming a full testing increase because of decentralization, operational model 3 (decentralization of testing to largest 20% of healthcare facilities) neared cost-effectiveness at less than $1000 per DALY averted across all testing distributions. If no downtime of decentralized instruments were assumed, operational model 3 would be considered cost-effective for healthcare systems that have high- and medium-density testing (Table S8).

### Threshold and sensitivity analysis

**Fig. 1:**
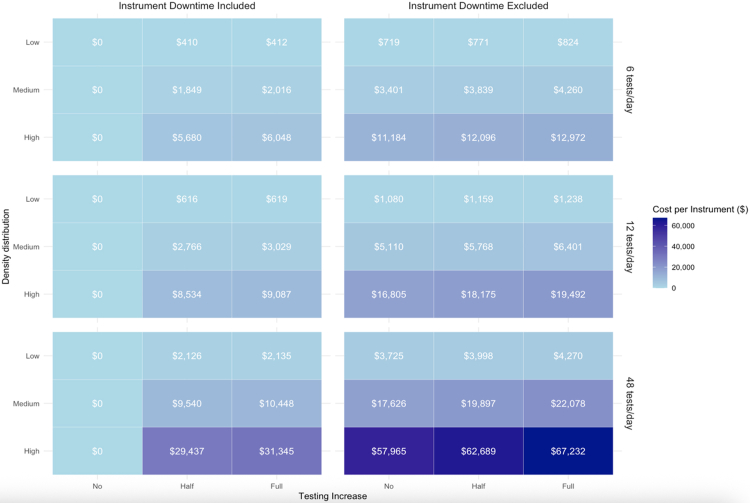
**Instrument and warranty prices to achieve cost-effectiveness at $500 per disability adjusted life-year (DALY) averted, with and without instrument downtime, at different instrument capacities.** The left panels includes 30-day downtime for decentralized testing and the right panel excludes instrument downtime. Results are stratified by the facility density distributions, instrument type, and testing increase (no, partial, and full increase).

We evaluated the price points required for instrument + warranty costs to make fully decentralized testing cost-effective at a $500 per DALY averted threshold across the three facility distributions. When instrument downtime is included—resulting in fully decentralized testing falling below $500/DALY averted—maximum instrument + warranty price ranges from $0 (“instrument-free”) when no testing increase is assumed (given the decrease in overall DALYs expected), to $410—$6048 for instruments can that do up to 6 tests/day ($616—$9087 for instruments that can do 12 tests/day) with a partial testing or full testing increase assumed (Fig. 1). When instrument downtime is excluded, the maximum price for instrument + warranty increases—$719–$11,184 for instruments that can do 6 tests/day ($1080—$16,804 for instruments that can do up to 12 tests/day). These costs only increased marginally when increased testing was expected as a result of decentralization.

The maximum price of an instrument also strongly depends on the density of healthcare facilities. In countries with low-density testing, the maximum price of instrument + warranty for instruments that can process up to 6 tests per day is $824 across all scenarios, and for instruments that can process up to 12 tests/day is $1238 across all scenarios (Fig. 1). For medium- and high-density testing settings, the instrument + warranty prices can be 4–5× higher and 14–16× higher respectively.

**Fig. 2:**
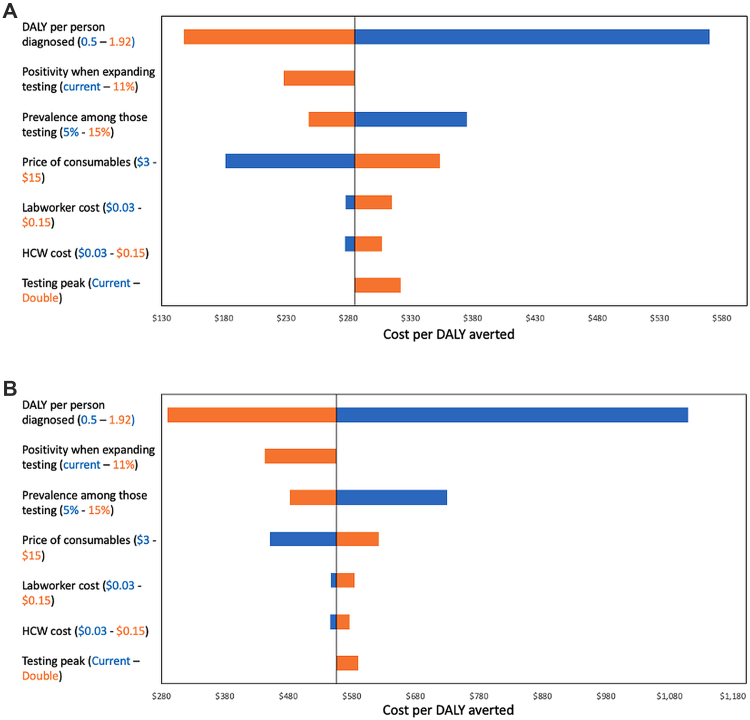
Tornado diagram of the impact of key input parameters on the average cost per disability adjusted life-year (DALY) averted: (A) partial testing increase with a high-density testing distribution, and (B) partial testing increase with a low-density testing distribution.

Our sensitivity analysis showed that the cost per DALY averted was most sensitive to the assumed number of DALYs averted per person diagnosed (Fig. 2). Increasing this value from 1 to 1.92 reduced the cost per DALY averted by 48% (from $285 to $148 per DALY averted in high-density settings), while decreasing it to 0.5 doubled the cost per DALY averted. Other influential parameters included: (1) underlying TB prevalence, with lower prevalence associated with higher costs per DALY averted, and (2) the price of consumables, with lower prices reducing costs per DALY averted. Similar trends were observed in low-density settings.

Determinants of incremental cost-effectiveness differed between high- and low-density settings (Figure S1). In low-density settings, specimen transport costs had no impact on the ICER of fully decentralized testing compared to the next least costly option. In contrast, in high-density settings, higher specimen transport costs were associated with a lower ICER for fully decentralized testing (38% decrease to $1218), whereas lower transport costs resulted in a higher ICER (28% increase to $2509). As with the average cost per DALY averted, both the assumed DALYs averted per person diagnosed and the underlying TB prevalence strongly influenced ICERs. The price of consumables and the extent of peak testing demand were moderately influential in high-density settings but had no effect in low-density settings. Human resource costs (healthcare worker and laboratory time) had minimal impact on either average or incremental cost-effectiveness.

## Discussion

The instruments modeled in our primary analysis are costly, as instrument + warranty prices currently range between $17,530–$90,854.^20^ At these prices, fully decentralized testing averted up to 23% more DALYs than centralized testing, but was not cost-effective under a $500/DALY averted threshold once instrument downtime was considered. Partial decentralization to the largest 20% of facilities could be cost-effective in medium- and high-density settings if downtime were minimized and results were returned at the same clinical encounter. Alternative molecular platforms such as Molbio's Trunat MTB RIF, priced at a lower $10,000–$18,000 (including one year of warranty),^29^ may change this picture. Lower instrument costs expand the scenarios where decentralized testing becomes cost-effective under our model assumptions, although trade-offs exist: while Xpert detects rifampicin resistance, other platforms may detect a broader range of resistance or none at all, necessitating reflex testing and potentially higher operational costs. These differences highlight how platform choice interacts with both cost and functionality in determining value for money.

Our threshold analysis shows that for decentralized testing to meet a $500/DALY averted benchmark, instrument and warranty costs must fall substantially: to $410–$824 for low volume instruments and $616–$1238 for medium volume instruments in low-density settings, and to 4–5 times and 14–16 times higher in medium- and high-density settings, respectively. These thresholds provide clear pricing targets for manufacturers and funders. New pipeline instruments, projected at $100–$2000 with per-test costs of $2–3^30^^,^^31^, could make decentralization cost-effective even in low-density contexts, particularly if downtime is eliminated. Greater instrument utilization, whether through increased TB demand or cross-utility for HIV, hepatitis B, or COVID-19, would further improve cost-effectiveness and strengthen the case for decentralized molecular platforms as part of universal health coverage objectives.

To the best of our knowledge, this is one of several studies that has looked at the cost-effectiveness of decentralizing TB testing, but is the first modeling study that aims to evaluates optimal instrument pricing as well as examining cost-effectiveness of decentralization of TB testing across a whole healthcare system (while accounting for changes to patient/sample loss during the diagnostic cascade).^32^ Previous studies looking at decentralization of GeneXpert for tuberculosis diagnosis have found it to be either cost-effective or cost-saving.^13^, ^14^, ^15^ Across both TB diagnostics, and other molecular tests such as HIV viral load, studies have found that instrument utilization is a large driver of cost-effectiveness for decentralized testing.^13^, ^14^, ^15^, ^16^^,^^33^ An early study by Sohn and colleagues^15^ found ICERs (assuming no cost sharing for specimen transport, as in our study) ranging from cost-saving at higher testing volumes to $2339 per DALY averted at lower volumes. While Sohn et al. evaluated a single testing volume, we modeled a distribution of volumes across facilities to simulate cost-effectiveness across a healthcare system. Nevertheless, the trends in cost-effectiveness are consistent between studies. Malhotra and colleagues,^13^ in the TB-CAPT trial of Truenat true point-of-care testing versus hub-and-spoke GeneXpert in Mozambique and Tanzania, estimated incremental costs per new treatment initiation of $422 and $580, respectively. While these are moderately lower than our estimates, this likely reflects trial-based evaluation rather than system-wide implementation. Similarly, Thompson and colleagues,^14^ in the XPEL-TB study of decentralized versus centralized testing, reported an additional cost per tuberculosis diagnosis of $1332 (range $763–$5558), well within the range of our findings.

This study has several limitations. Firstly, our modeling focused exclusively on sputum-based sample types and the DALY associated with this specimen use. Notably the inclusion of diverse sample types, such as swabs, may increase testing demand. This stems from the ease of sample provision (particularly for individuals living with HIV) and the increased likelihood of providers offering tests.^34^ Analytically, this potential increase in testing is likely to be captured within the different rates of testing demand we have already evaluated- making the partial or full testing increase scenarios even more likely if swabs become the specimen of choice. Second, we did not include the cost of specimen storage at centralized facilities during downtime, which may increase operational model costs marginally. Furthermore, our costing of clinical encounters was limited to staff time directly associated with testing. Other costs such as administrative processes and consultations were not included, as they would remain the same across scenarios. Third, we used three facility density distributions to reflect differences in healthcare systems across high-burden TB countries globally (Table S1). While we aimed to capture a wide range of settings, this analysis may not fully represent all high-burden TB areas. The trends of our analysis can be extrapolated in either direction. In healthcare systems with greater testing demand per facility (related to <1000 facilities for 500,000 annual tests in our analysis), decentralization of testing is even more cost-effective given the expected higher utilization per instrument. At lower-density healthcare systems than those evaluated (>5000 facilities for 500,000 annual tests), decentralization is not cost-effective at the $500/DALY averted threshold. Fourth, We assumed a positivity rate of 11% reflecting positivity rates in high TB burden countries. When demand increases substantially, observed positivity rates may decline. To account for this, we used estimates from a recent trial that demonstrated an 8% positivity rate when TB testing was decentralized. Further relative decreases in positivity rate may reduce the cost-effectiveness point estimate of decentralized testing as compared to centralized testing, which we have explored in our sensitivity analysis. However, if the instrument prices outlined in this article can be achieved, fully decentralized testing is likely to be cost-effective across many settings. Fifth, we assumed that each TB diagnosis averts 1 DALY, consistent with estimates in the literature (range: 0.5–1.92 DALYs per diagnosis).^26^^,^^27^ This value provides a reasonable, and possibly conservative, midpoint assumption. If the true DALYs averted per diagnosis are higher than our base case, threshold prices for point-of-care instruments and the cost-effectiveness of decentralized testing would correspondingly increase. For example, if 5 DALYs averted per diagnosis were assumed- the relative cost-effectiveness of decentralized testing would be substantially greater than reported here. By adopting a conservative estimate, our analysis errs on the side of under- rather than over-stating potential benefits, which is important for pricing: if the benefits were overestimated, instruments could be priced too high relative to their actual value, whereas underestimation ensures that any future evidence of greater benefit would only strengthen the economic case. Finally, we assumed instruments are out of service 30 days per year.^7^ However, this estimate is based on a decentralized TB molecular testing. If testing is fully decentralized, instrument downtime may be greater given the increased number of instruments to service, along with the diverse, and often remote, geographies that need to be reached. If downtime is greater than assumed in our analysis, this could result in worse health outcomes than a centralized scenario; therefore, monitoring instrument downtime as testing decentralizes to ensure and preserve gains in health outcomes is essential.

Given the possible expected improvements in diagnostic yield and related downstream health benefits, decentralization of TB testing should be considered. However, the extent to which decentralization should be implemented depends strongly on the volume of tests, price of the instrument and the downtime expected, with full decentralization only recommended under significantly reduced instrument + warranty prices and downtime ≤30 days guaranteed. Through these results, and from prior studies, it is likely that a partially decentralized approach would yield the greatest balance in terms of cost and performance (new diagnoses), and a fully decentralized approach may be more feasible with lower instrument prices. Future work, especially as new instruments release to market, should focus on understanding what the most beneficial level of decentralization may be in different country contexts. Further, as these new tests and platforms are developed, this study provides manufactures and healthcare programmes with guidance on cost-effective target prices for different implementation scenarios and settings. This would provide evidence to support price negotiations between developers and countries to balance healthcare resource requirements while remaining a viable development option for manufacturers.

In conclusion, this study highlights the complexities surrounding the decentralization of TB molecular testing. While decentralization increased diagnostic yield and averts DALYs, under current instrument-warranty costs, full decentralization is unlikely to be cost-effective. Partial decentralization may be cost-effective if demand for testing is simultaneously increased and limited downtime can be guaranteed. Sustainable decentralization of testing requires that instrument prices be reduced significantly, with greater reductions required for low-density test settings. The target price points provided in this analysis could be used to inform guide manufacturers on their development strategies, by indicating the type and cost of tests most useful to health programs in high-TB burden settings.

## Contributors

BEN conceived and designed the analysis. TO performed the analysis. BEN and TO accessed and verified the data. All authors participated in interpreting the results. TO, AdN, BEN wrote the first draft of the paper. All authors reviewed and edited the paper. All authors decided to publish.

## Data sharing statement

Analysis can be replicated using the assumptions, data and equations defined in the main text and Supplementary Materials.

## Declaration of interests

The authors have no conflicts of interest to declare.
