# Supplementary material for: Cost and impact of decentralized tuberculosis testing: a modeling analysis of price thresholds for molecular instruments in high-burden settings
Source: eClinicalMedicine. 2025 Dec 29;91:103728. doi: 10.1016/j.eclinm.2025.103728 (PMC12803841; doi:10.1016/j.eclinm.2025.103728)
Supplement: Supplementary Material [file mmc1.docx]

**Supplementary Materials**

**Text S1.** Decentralization scenario descriptions

*Centralized*: All testing is conducted at a single centralized site equipped with GeneXpert instruments. Individuals seeking care must return to the healthcare facility to obtain their test results. Potential LTFU factors are considered, including the loss of specimens during transportation to the centralized facility.

*Decentralized molecular testing on-site*: GeneXpert instruments are deployed at some or all healthcare facilities (scenario dependent). The instrument assigned to each healthcare facility (either 2-module, 4-module or 16-module GeneXpert instrument) depended on the expected number of tests to be conducted at that site. These operational models facilitate on-site testing for all individuals, thereby eliminating sample loss during transportation. It is still assumed that all individuals seeking care are required to return to the healthcare facility to collect their test results.

*Decentralized molecular testing on-site- same-clinical-encounter*: In the same-clinical-encounter decentralized testing operational model, we implement a minimum number of GeneXpert instruments to facilitate same-clinical-encounter results. This eliminates the need for individuals to return to the healthcare facility to collect their result and initiate treatment (if needed). Given the distribution of individuals seeking care in a day, more instruments may be required than in the decentralized on-site operational model to ensure results are returned within the same clinic visit.

For this operational model, healthcare utilization data on specimen provision was used^1^. In many countries without an appointment-based care system, most individuals seek or receive care between 8-10am. We calculated the proportion of individuals offered testing within a specific timeframe as a fraction of the total number of individuals seeking care throughout the entire day^1^. We then allocated the appropriate instrument size (2-, 4-, or 16- module) to the healthcare facility tailored to accommodate peak testing volume during this timeframe.

**Table S1. Distribution of patient blood draws by time of day.** This table summarizes the number and proportion of patient blood draws conducted across hourly intervals from 7:00 AM to 4:00 PM in South Africa.^1^

| **N** | **Time** | **Patient blood draw distribution** | **Proportion of samples taken** |
| --- | --- | --- | --- |
| 2 | 7-8am | 3.80% |  |
| 12 | 8-9am | 22.60% | 26% |
| 13 | 9-10am | 24.50% | 51% |
| 9 | 10-11am | 17.00% | 68% |
| 8 | 11-12pm | 15.10% | 83% |
| 2 | 12-1pm | 3.80% | 87% |
| 2 | 1-2pm | 3.80% | 91% |
| 4 | 2-3pm | 7.50% | 98% |
| 1 | 3-4pm | 1.90% | 100% |
| 53 |  | 100% |  |

**Text S2.** Description of main cost components

*Capital costs:* Includes the cost of instruments, warranties, and maintenance for each type of instrument (2-,4- and 16-module). Costs were sourced from the StopTB Global Drug Fund catalogue^2^ and annualized based on an assumed useful life of 8 years for instruments, and 3 years for warranty and maintenance. Daily costs were estimated by dividing the annual cost by 260 working days.

*Reagents and consumables:* Includes the cost of consumables required for molecular testing as well as those needed for sputum sample collection^2^ adjusted to 2024 USD.

*Staff time:* Assumptions were made regarding the hands-on time by staff to collect a sputum sample and perform the molecular tests. Activities were performed by nurses and laboratory technicians. To maintain a country-agnostic perspective while focusing on high-burden settings, we used publicly available salary data from a selection of high-TB-burden countries. These salaries were averaged to estimate the general staff cost per minute and multiplied by the time required for each activity.

*Overhead costs:* Includes overhead costs per test stratified by centralized and decentralized testing, sourced from existing literature^3^.

*Transport costs:* Transportation costs were calculated as a percentage of the total test cost and varied by operational model, sourced from the literature (Table S2)^4^.

**Table S2.** Total number of healthcare facilities, annual number of TB test, and total number of TB tests per facility stratified by country.

| Country | Total number of healthcare facilities | Total number of annual TB tests | Total number of tests per facility | Reference |
| --- | --- | --- | --- | --- |
| Zambia | 2,500 | 400,000 | 160 | ^5^ |
| South Africa | 4,000 | 2,000,000 | 500 | ^6,7^ |
| Indonesia | 10,000 | 2,000,000 | 200 | ^5,8^ |

**Text S3**. Model equations

For facilities in the healthcare systems of different testing densities, let facilities be *i*=1,..,*N* and operational models *s*∈{1,…,5}.

***Facility demand.*** Facility demand is defined as the baseline annual test demand at facility *i* using Pareto size distribution:

(Equation 1): $D_{i}\sim Pareto\left( x_{min},\alpha\right), D_{i}\geq x_{min},\alpha>1$

The demand $D_{i}$ represents the number of tests per year that facility *i* would provide under a centralized testing system and serve as the fixed starting point for all subsequent equations.

***Operational model allocation*** (centralized vs decentralized). Facilities are assigned to either centralized or decentralized testing depending on the operational model *s*. We define an allocation indicator of:

(Equation 2)

$$a_{i,s}=\left\{ \begin{aligned} 1, \mathrm{facility}i is decentralized in operational model s \\ 0, \mathrm{facility}i is centralized in operational model s \end{aligned} \right.$$

Here, *s* indexes the operational model. For example, model 1 corresponds to “fully centralized” where all $a_{i,1}=0$. Model 4 and model 5 correspond to “fully decentralized,” where all $a_{i,1}=1$. Intermediate models (e.g., model 2 and model 3) allow a mix of centralized and decentralized facilities. $a_{i,s}$ is always binary, and the operational model index *s* determines the overall mix of centralized versus decentralized facilities.

***Expansion and downtime*.** Decentralization may increase demand because more individuals can be tested when diagnostics are placed closer to individuals seeking care. We capture this using an expansion factor $m_{s}\geq1$, which scales up baseline demand at decentralized facilities. At the same time, decentralized placement may lead to more frequent downtime of machines due to operational challenges, supply chain interruptions, and difficult in servicing a large number of instruments even in difficult to reach places. We denote this as $d_{s}\in\left[ 0,Y \right]$ days of downtime per year, where *Y=365*. For centralized facilities, $d_{s}=0$.

The decentralized availability factor is then:

(Equation 3): $q_{s}=1-\frac{d_{s}}{Y}\in[0,1]$

Thus, increased demand from decentralization ($m_{s}$) is offset by higher downtime ($d_{s}$), which reduces the effective availability of decentralized instruments.

***Effective number of tests performed*.** The number of tests performed at facility *i* in operational model *s* is:

(Equation 4): $T_{i,s}=D_{i}[\left( 1-a_{i,s} \right)+a_{i,s}m_{s}q_{s}]$

For centralized facilities ($a_{i,s}=0)$, demand remains $D_{i}$.

For decentralized facilities ($a_{i,s}=1)$, demand is scaled by $m_{s}$ and reduced by downtime via $q_{s}$.

This ensures the model explicitly reflects the trade-off between increased reach through decentralization and reduced throughput due to downtime.

***Test positivity*.** Let $\rho_{s}^{C}$ be the baseline true positivity among those tested under centralized testing. For decentralized testing, positivity may differ due to differences in populations reached. We therefore define:

(Equation 5): $\rho_{s}^{D}=\left\{ \begin{aligned} \rho_{s}^{C}, m_{s}=1 \\ \rho_{s}^{expected},m_{s}>1 \end{aligned} \right.$

Then, the positivity at facility *i* in operational model *s* is then:

(Equation 6): $\rho_{i,s}=\left( 1-a_{i,s} \right)\rho_{S}^{C}+a_{i,s}\rho_{S}^{D}$

This formulation allows positivity to remain unchanged when decentralization does not expand testing ($m_{s}=1)$, but to shift if expansion reaches populations with different prevalence.

***Test sensitivity*.** Incorporating test sensitivity (with , $Se_{s}^{C} \mathrm{and}Se_{s}^{D}$ defined separately in case of differing test sensitivities in future model iterations) let:

(Equation 7): $Se_{i,s}=\left( 1-a_{i,s} \right)Se_{s}^{C}+ a_{i,s}Se_{s}^{D}$

***Loss to follow-up.*** We allow for differences in loss to follow-up depending on whether results are returned in the same clinical encounter (decentralized) or not (centralized). The facility-level loss to follow-up is defined as:

(Equation 8): $l_{i,s}=\left( 1-a_{i,s} \right)l_{C}+a_{i,s}l_{D}$

where $l_{C}$is the centralized LTFU rate, $l_{D}$the decentralized LTFU rate, and typically $l_{D}< l_{C}$.

***Positives detected and linked.*** Then, the total number of positives detected is expressed as:

(Equation 9): $P_{i,s}=T_{i,s}\rho_{i,s}$

And those successfully linked and treated as:

(Equation 10): $L_{i,s}=P_{i,s}(1-l_{i,s})$

***Aggregated totals.*** Finally, we calculate operational model totals by summing across facilities:

1. $T_{s}=\sum_{i=1}^{N} T_{i,s}$
2. $P_{s}=\sum_{i=1}^{N} P_{i,s}$
3. $L_{s}=\sum_{i=1}^{N} L_{i,s}$

**Table S3.** Cost parameters – assumptions and sources.

| Parameter | Estimate | Source and assumptions |
| --- | --- | --- |
| Transport cost | | |
| Transport cost per test | Operational model 1: 18.5% of total test cost  Operational model 2 & 3: 31% of total test cost  Operational model 4 & 5: 0% | Transportation costs were calculated as a percentage of the total test cost and varied by operational model^4^ |
| Instrument and warranty costs | | |
| GeneXpert II-module | $13,030.00 | 8 working life years assumed^2^ |
| GeneXpert IV-module | $19,500.00 | 8 working life years assumed^2^ |
| GeneXpert XVI-module | $72,350.00 | 8 working life years assumed^2^ |
| GeneXpert II warranty | $4,500.00 | Warranty spanning 3 years^2^ |
| GeneXpert IV warranty | $6,840.00 | Warranty spanning 3 years^2^ |
| GeneXpert XVI warranty | $18,504.00 | Warranty spanning 3 years^2^ |
| Consumable cost | | |
| GeneXpert MTB/RIF kit 50 tests | $398.50 | Price included kit of 50 tests^2^ |
| Staff cost | | |
| Staff cost nurse | $0.06/min | Average nurse salary per minute calculated using publicly available salary data from five low- and middle-income countries (Kenya, Zambia, South Africa, India, and Vietnam), based on an assumption of 260 working days per year^9–13^. |
| Staff cost laboratory technician | $0.05/min | Average laboratory technician salary per minute calculated using publicly available salary data from five low- and middle-income countries (Kenya, Zambia, South Africa, India, and Vietnam), based on an assumption of 260 working days per year ^14–18^ |
| Overhead cost | | |
| Decentralized cost | $0.51 per test | ^3^ |
| Centralized cost | $0.60 per test | ^3^ |

**Table S4.** Cost per sample transported, data from sample transport optimization in Zambia^4^

|  | Cost of the test excluding specimen transport (viral load for this example) | Cost including specimen transport | Percent of the test related to specimen transport costs |
| --- | --- | --- | --- |
| Specimens transported from 100% of facilities to centralized facilities (fully centralized system) | 17.22 | 21.13 | 18.5% |
| Transporting specimens to 80% of smallest facilities | 17.22 | 22.55 | 23.6% |
| All equipment full decentralized | 17.22 | 0 | 0% |

**Table S5.** Breakdown of cost categories and total cost per test across operational models when assuming no increase in testing uptake. Costs include equipment, consumables, staff, transport, and overhead, with variations driven by operational models (centralized vs decentralized and facility level).

| High-density testing distribution | | | | | |  |
| --- | --- | --- | --- | --- | --- | --- |
| Cost category | Operational model 1 | Operational model 2 | Operational model3 | Operational model 4 | Operational model 5 | |
| Equipment | $1.35 | $2.49 | $3.64 | $7.60 | $8.74 | |
| Consumables | $10.28 | $10.28 | $10.28 | $10.28 | $10.28 | |
| Staff | $2.27 | $2.27 | $2.27 | $2.27 | $2.27 | |
| Transport | $3.29 | $2.11 | $2.26 | $0 | $0 | |
| Overhead | $0.60 | $0.54 | $0.54 | $0.51 | $0.51 | |
| Total cost per test | $17.79 | $17.68 | $18.98 | $20.66 | $21.80 | |
| Medium-density testing distribution | | | | | |  |
| Equipment | $1.35 | $3.66 | $4.73 | $14.43 | $15.50 | |
| Consumables | $10.28 | $10.28 | $10.28 | $10.28 | $10.28 | |
| Staff | $2.27 | $2.27 | $2.27 | $2.27 | $2.27 | |
| Transport | $3.29 | $1.39 | $1.48 | $0 | $0 | |
| Overhead | $0.60 | $0.53 | $0.53 | $0.51 | $0.51 | |
| Total cost per test | $17.79 | 18.13 | $19.29 | $27.49 | $28.56 | |
| Low-density testing distribution | | | | | |  |
| Equipment | $1.35 | $7.63 | $8.65 | $35.08 | $36.10 | |
| Consumables | $10.28 | $10.28 | $10.28 | $10.28 | $10.28 | |
| Staff | $2.27 | $2.27 | $2.27 | $2.27 | $2.27 | |
| Transport | $3.29 | $0.80 | $0.84 | $0 | $0 | |
| Overhead | $0.60 | $0.52 | $0.52 | $0.51 | $0.51 | |
| Total cost per test | $17.79 | $21.50 | $22.56 | $48.14 | $49.16 | |

* Decentralized operational models have higher equipment costs, resulting in higher total costs per test.

**Table S6*.*** Breakdown of cost categories and total cost per test across operational models when assuming a half increase in testing uptake. Costs include equipment, consumables, staff, transport, and overhead, with variations driven by operational models (centralized vs decentralized and facility level).

| High-density testing distribution | | | | | |  |
| --- | --- | --- | --- | --- | --- | --- |
| Cost category | Operational model 1 | Operational model 2 | Operational model3 | Operational model 4 | Operational model 5 | |
| Equipment | $1.35 | $2.37 | $3.49 | $6.11 | $7.28 | |
| Consumables | $10.28 | $10.28 | $10.28 | $10.28 | $10.28 | |
| Staff | $2.27 | $2.27 | $2.27 | $2.27 | $2.27 | |
| Transport | $3.29 | $1.69 | $1.81 | $0 | $0 | |
| Overhead | $0.60 | $0.53 | $0.53 | $0.51 | $0.51 | |
| Total cost per test | $17.79 | $17.14 | $18.38 | $19.17 | $20.34 | |
| Medium-density testing distribution | | | | | |  |
| Equipment | $1.35 | $3.20 | $4.36 | $11.30 | $12.41 | |
| Consumables | $10.28 | $10.28 | $10.28 | $10.28 | $10.28 | |
| Staff | $2.27 | $2.27 | $2.27 | $2.27 | $2.27 | |
| Transport | $3.29 | $1.08 | $1.15 | $0 | $0 | |
| Overhead | $0.60 | $0.53 | $0.53 | $0.51 | $0.51 | |
| Total cost per test | $17.79 | $17.35 | $18.59 | $24.36 | $25.47 | |
| Low-density testing distribution | | | | | |  |
| Equipment | $1.35 | $6.19 | $7.28 | $27.05 | $28.11 | |
| Consumables | $10.28 | $10.28 | $10.28 | $10.28 | $10.28 | |
| Staff | $2.27 | $2.27 | $2.27 | $2.27 | $2.27 | |
| Transport | $3.29 | $0.59 | $0.62 | $0 | $0 | |
| Overhead | $0.60 | $0.52 | $0.52 | $0.51 | $0.51 | |
| Total cost per test | $17.79 | $19.85 | $20.97 | $40.11 | $41.17 | |

*Decentralized operational models have higher equipment costs, resulting in higher total costs per test.

**Table S7.** Breakdown of cost categories and total cost per test across operational models when assuming a full increase in testing uptake. Costs include equipment, consumables, staff, transport, and overhead, with variations driven by operational models (centralized vs decentralized and facility level).

| High-density testing distribution | | | | | |  |
| --- | --- | --- | --- | --- | --- | --- |
| Cost category | Operational model 1 | Operational model 2 | Operational model3 | Operational model 4 | Operational model 5 | |
| Equipment | $1.35 | $2.25 | $3.35 | $4.62 | $5.82 | |
| Consumables | $10.28 | $10.28 | $10.28 | $10.28 | $10.28 | |
| Staff | $2.27 | $2.27 | $2.27 | $2.27 | $2.27 | |
| Transport | $3.29 | $1.27 | $1.36 | $0 | $0 | |
| Overhead | $0.60 | $0.53 | $0.53 | $0.51 | $0.51 | |
| Total cost per test | $17.79 | $16.60 | $17.78 | $17.68 | $18.88 | |
| Medium-density testing distribution | | | | | |  |
| Equipment | $1.35 | $2.74 | $3.99 | $8.17 | $9.31 | |
| Consumables | $10.28 | $10.28 | $10.28 | $10.28 | $10.28 | |
| Staff | $2.27 | $2.27 | $2.27 | $2.27 | $2.27 | |
| Transport | $3.29 | $0.76 | $0.82 | $0 | $0 | |
| Overhead | $0.60 | $0.52 | $0.52 | $0.51 | $0.51 | |
| Total cost per test | $17.79 | $16.57 | $17.88 | $21.23 | $22.37 | |
| Low-density testing distribution | | | | | |  |
| Equipment | $1.35 | $4.76 | $5.91 | $19.01 | $20.12 | |
| Consumables | $10.28 | $10.28 | $10.28 | $10.28 | $10.28 | |
| Staff | $2.27 | $2.27 | $2.27 | $2.27 | $2.27 | |
| Transport | $3.29 | $0.38 | $0.40 | $0 | $0 | |
| Overhead | $0.60 | $0.52 | $0.52 | $0.51 | $0.51 | |
| Total cost per test | $17.79 | $18.20 | $19.38 | $32.07 | $33.18 | |

*Decentralized operational models have higher equipment costs, resulting in higher total costs per test.

**Table S8.** Daily costs, DALYs averted and ICERs across key operational models, facility density distribution and various testing uptake scenarios (no testing increase, partial increase, or full increase) without instrument downtime. Operational models reflect differences in costs and health outcomes, with more decentralized models (models 3 and 5) incurring higher costs but achieving greater DALYs averted. ICER values highlight the additional cost per DALY averted when moving between models.

| No testing increase | | | | Partial testing increase | | | | Full testing increase | | | |
| --- | --- | --- | --- | --- | --- | --- | --- | --- | --- | --- | --- |
| Operational model | Cost (daily) | DALYs averted (daily) | ICER | Scenario | Cost (daily) | DALYs averted (daily) | ICER | Scenario | Cost (daily) | DALYs averted (daily) | ICER |
| High-density testing distribution | | | | | | | | | | | |
| 1 | $30,924 | 179 | Ref | 1 | $30,924 | 179 | Ref | 1 | $30,924 | 179 | Ref |
| 2 | $31,333 | 182 | D | 2 | $40,355 | 201 | $387.2 | 2 | $49,379 | 221 | $423.8 |
| 3 | $33,640 | 187 | $470.9 | 3 | $43,273 | 208 | $508.4 | 3 | $52,906 | 228 | $537.20 |
| 4 | $39,723 | 184 | D | 6 | $52,156 | 211 | WD | 6 | $64,589 | 239 | WD |
| 5 | $41,931 | 191 | $1,441.3 | 7 | $55,459 | 220 | $753.6 | 7 | $68,987 | 249 | $630.6 |
| Medium-density testing distribution | | | | | | | | | | | |
| 1 | $30,924 | 179 | Ref | 1 | $30,924 | 179 | Ref | 1 | $30,924 | 179 | Ref |
| 2 | $33,104 | 183 | $164.5 | 2 | $43,336 | 205 | $409.6 | 2 | $53,568 | 228 | $429.6 |
| 3 | $35,216 | 189 | $359.4 | 3 | $46,512 | 212 | $463.2 | 3 | $57,807 | 236 | $543.1 |
| 4 | $52,874 | 184 | D | 6 | $65,225 | 211 | D | 6 | $77,575 | 239 | WD |
| 5 | $54,930 | 191 | $7,698.6 | 7 | $68,338 | 220 | $2,617.8 | 7 | $81,747 | 249 | $1,709.2 |
| Low-density testing distribution | | | | | | | | | | | |
| 1 | $30,924 | 179 | Ref | 1 | $30,924 | 179 | Ref | 1 | $30,924 | 179 | Ref |
| 2 | $40,321 | 183 | WD | 2 | $51,619 | 208 | WD | 2 | $62,917 | 234 | WD |
| 3 | $42,310 | 190 | $801.1 | 3 | $54,653 | 216 | $569.0 | 3 | $66,996 | 243 | $527.4 |
| 4 | $92,575 | 184 | D | 6 | $104,882 | 211 | D | 6 | $117,189 | 239 | D |
| 5 | $94,538 | 191 | $47,547.8 | 7 | $107,888 | 220 | $14,729.9 | 7 | $121,238 | 249 | $8,860.6 |

D- dominated (more costly and averts fewer DALYs than the next least costly scenario on the cost-effectiveness frontier)

WD- weakly dominated (not on the cost-effectiveness frontier)

**Figure S1.** Tornado diagram of the impact of key input parameters on the incremental cost per DALY averted: (A) partial testing increase with a high-density testing distribution, and (B) partial testing increase with a low-density testing distribution.


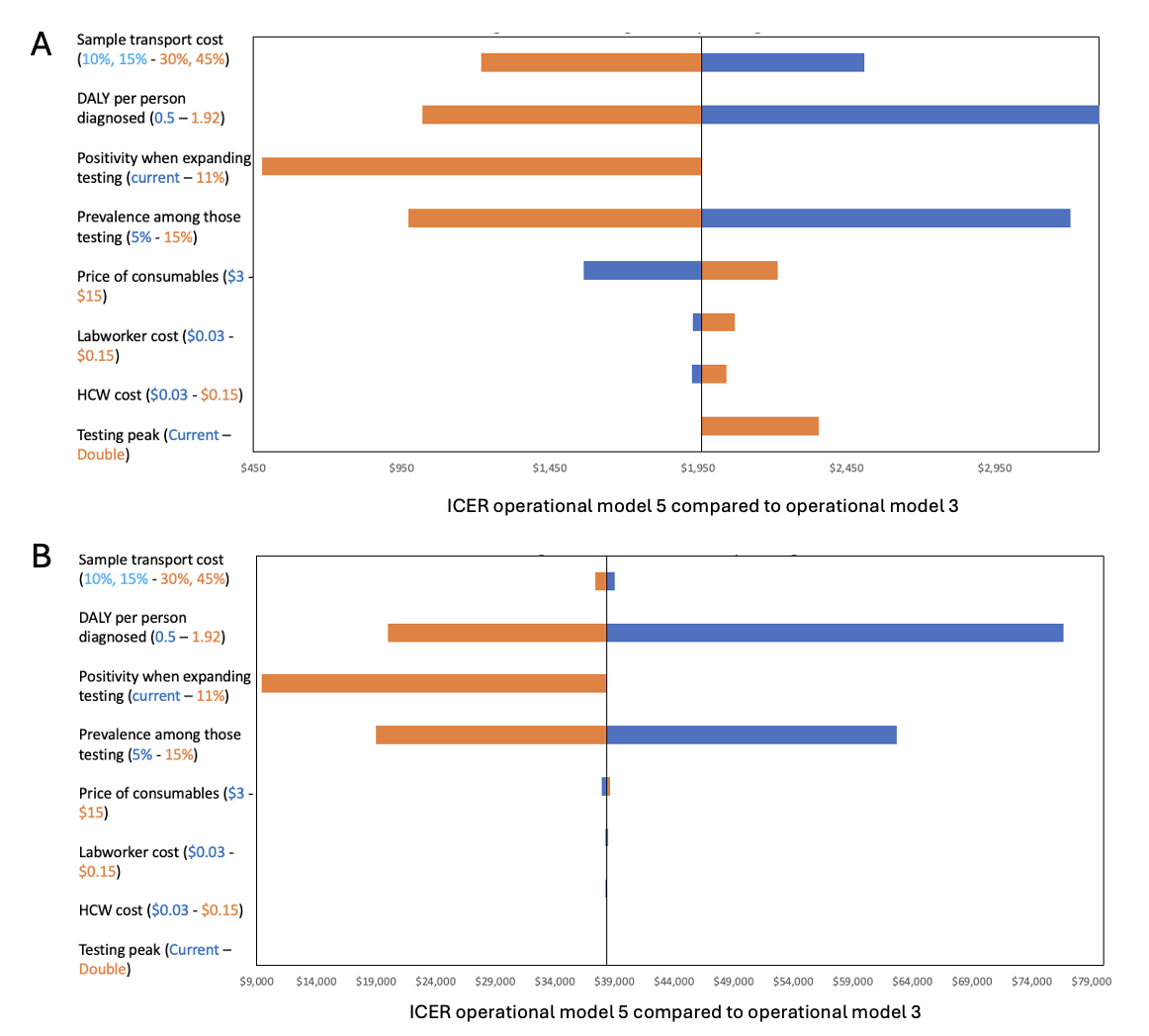


**References**

1 Girdwood SJ, Crompton T, Cassim N, *et al.* Optimising courier specimen collection time improves patient access to HIV viral load testing in South Africa. *Afr J Lab Med* 2022; **11**: 1725.

2 GDF Product Catalog | Stop TB Partnership. https://www.stoptb.org/global-drug-facility-gdf/gdf-product-catalog (accessed April 18, 2024).

3 Girdwood SJ, Nichols BE, Moyo C, Crompton T, Chimhamhiwa D, Rosen S. Optimizing viral load testing access for the last mile: Geospatial cost model for point of care instrument placement. *PLoS One* 2019; **14**: e0221586.

4 Nichols BE, Girdwood SJ, Crompton T, *et al.* Monitoring viral load for the last mile: what will it cost? *Journal of the International AIDS Society* 2019; **22**: e25337.

5 Data. https://www.who.int/teams/global-tuberculosis-programme/data (accessed Jan 13, 2025).

6 South African National AIDS Council. SANAC. https://sanac.org.za/ (accessed Jan 13, 2025).

7 Africa SS. Health | Statistics South Africa. 2024; published online Aug 27. https://www.statssa.gov.za/?cat=27 (accessed Jan 13, 2025).

8 Welcome To Ministry of Health Republic Indonesia. https://kemkes.go.id/eng/home (accessed Jan 13, 2025).

9 Registered Nurse (RN) Salary in Kenya in 2025 | PayScale. https://www.payscale.com/research/KE/Job=Registered_Nurse_(RN)/Salary (accessed Aug 19, 2025).

10 Registered nurse salary in South Africa. https://za.indeed.com/career/registered-nurse/salaries (accessed Aug 19, 2025).

11 Registered Nurse (RN) Salary in Zambia in 2025 | PayScale. https://www.payscale.com/research/ZM/Job=Registered_Nurse_(RN)/Salary (accessed Aug 19, 2025).

12 Registered Nurse Salary in India (2025). https://www.erieri.com/salary/job/registered-nurse/india (accessed Aug 19, 2025).

13 Registered Nurse Salary in Vietnam (2025). https://www.erieri.com/salary/job/registered-nurse/vietnam (accessed Aug 19, 2025).

14 Lab Technologist Salary in Kenya in 2025 | PayScale. https://www.payscale.com/research/KE/Job=Lab_Technologist/Salary (accessed Aug 19, 2025).

15 Average Lab Technician Salary in Zambia for 2025. World Salaries. https://worldsalaries.com/average-lab-technician-salary-in-zambia/ (accessed Aug 19, 2025).

16 Medical Laboratory Technician Salary in South Africa in 2025 | PayScale. https://www.payscale.com/research/ZA/Job=Medical_Laboratory_Technician/Salary (accessed Aug 19, 2025).

17 Medical Laboratory Technician Salary in India in 2025 | PayScale. https://www.payscale.com/research/IN/Job=Medical_Laboratory_Technician/Salary (accessed Aug 19, 2025).

18 Medical Laboratory Technician Salary in Vietnam (2025). https://www.erieri.com/salary/job/medical-laboratory-technician/vietnam (accessed Aug 19, 2025).
